# The Cwr1 protein kinase localizes to the plasma membrane and mediates resistance to cell wall stress in *Candida albicans*

**DOI:** 10.1128/msphere.00391-24

**Published:** 2024-11-29

**Authors:** Shamoon Naseem, Jakub Zahumenský, Carla E. Lanze, Lois M. Douglas, Jan Malínský, James B. Konopka

**Affiliations:** 1 Department of Microbiology and Immunology, Stony Brook University, Stony Brook, New York, USA; 2 Department of Functional Organization of Biomembranes, Institute of Experimental Medicine, Academy of Sciences of the Czech Republic, Prague, Czechia; University of Georgia, Athens, Georgia, USA

**Keywords:** C2_04360W, ORF19.4518, Ypl150w, eisosome, eisosomes, MCC domain, stress resistance, hyphal morphogenesis

## Abstract

The plasma membrane is critical for the virulence of the human fungal pathogen *Candida albicans*. In addition to functioning as a protective barrier, the plasma membrane plays dynamic roles in a wide range of functions needed for virulence including nutrient uptake, cell wall synthesis, morphogenesis, resistance to stress, and invasive hyphal growth. Screening a collection of *C. albicans* mutants identified an understudied gene that is important for invasive hyphal growth, which we have termed *CWR1* (Cell Wall Regulatory kinase). A mutant strain lacking *CWR1* displayed defects in resisting stressful conditions that exacerbate cell wall defects. The Cwr1 protein shows strong similarity to protein kinases, suggesting it plays a regulatory role in coordinating plasma membrane and cell wall functions. A Cwr1–green fluorescent protein (GFP) fusion protein localized to punctate patches associated with the plasma membrane that partially overlapped Membrane Compartment of Can1 (MCC)/eisosome domains. In contrast to the static MCC/eisosome domains, the Cwr1–GFP patches were very dynamic. Truncation mutants lacking C-terminal sequences distal to the protein kinase domain failed to show detectable localization at the plasma membrane. Surprisingly, these mutant strains did not show the defects of a *cwr1Δ* mutant, suggesting that localization to punctate patches associated with the plasma membrane is not essential for Cwr1 function. Altogether, these data indicate that Cwr1 contributes to the regulation of plasma membrane functions that promote proper morphogenesis and resistance to cell wall stress, both of which are important for *C. albicans* virulence.

**IMPORTANCE:**

The ability of *Candida albicans* to grow invasively in the host and resist stress is critical for it to be an effective human pathogen. Identifying the genes that promote these processes is important for developing new strategies to block infection. Therefore, genetic methods were used in this study to identify a novel gene that is needed for invasive growth and stress resistance (Cell Wall Regulatory kinase [*CWR1*]). Interestingly, the Cwr1 protein localized to punctate patches in the plasma membrane, some of which co-localized with specialized subdomains of the plasma membrane known as eisosomes that are known to promote stress resistance and invasive growth in the host. Thus, these studies identified a novel regulator of traits that are critical for *C. albicans* pathogenesis.

## INTRODUCTION

*Candida albicans* commonly grows as a harmless commensal organism on the human skin and mucosa. However, it can cause severe mucosal infections or lethal systemic infections when the immune system is impaired. *C. albicans* infections also commonly occur under conditions that promote an overgrowth of *C. albicans* that overwhelms the immune system, which can result from the use of antibacterial antibiotics that disrupt the microbiota or from biofilm formation on medical devices (1, 2). One of the reasons *C. albicans* is an effective pathogen is its ability to resist stressful conditions encountered in the host. This includes changes in nutrition, elevated temperature, cell wall stress, and attack by the immune system with oxidation, antimicrobial peptides, and other toxic conditions (3–5). Another factor that promotes virulence is the ability of *C. albicans* to switch from budding to filamentous hyphal growth, which mediates biofilm formation and invasive growth into tissues (6–10).

Genetic studies indicate that promoting proper cellular morphogenesis, invasive hyphal growth, and resistance to conditions that cause cell wall stress are inter-related processes. An interesting example of this interrelationship can be found in studies of the plasma membrane microdomains known as MCC (Membrane Compartment of Can1) or eisosomes that have been detected in fungi and unicellular algae, but have not been observed in other eukaryotic organisms (11, 12). In *C. albicans*, eisosomes have been shown to be important for promoting stress resistance and invasive hyphal growth (13–17). The MCC domains correspond to inward furrows in the plasma membrane (18), which are stabilized by a complex of cytosolic proteins termed the eisosome (19). There are approximately 50 of these domains per cell, each of which is approximately 300-nm long and 50-nm deep (19, 20). The Bin/amphiphysin/Rvs (BAR) domain containing proteins Pil1 and Lsp1 bind the plasma membrane and polymerize in a manner that forms the furrows (21–23). Studies with *C. albicans* and *Saccharomyces cerevisiae* have identified over 20 proteins that are associated with the cytoplasmic side of eisosomes, many of which contribute to morphogenesis and stress resistance (12, 24–27). Integral membrane proteins recruited into these domains include two distinct families of tetra-span proteins related to Sur7 and Nce102, which have been shown to be important for regulating morphogenesis and resisting cell wall stress (15, 28). Although the plasma membrane portion of these domains is known as MCC and the cytoplasmic proteins that associate on the intracellular side are known as eisosomes, for simplicity, we will refer to both structures as eisosomes.

We previously screened *C. albicans* deletion mutant collections for the ability to undergo invasive growth using a 48-pin replicator to transfer cells from 96-well plates to agar Petri plates containing different types of hyphal inducers. The strongest set of mutants was described previously (7). In this study, we decided to examine another mutant that has not been well studied that lacks a gene that encodes a protein with strong similarity to protein kinases (orf19.4518; C2_04,360W). One reason is that *S. cerevisiae* mutant cells lacking the ortholog of orf19.4518, known as YPL150W, showed increased sensitivity to certain types of stress in high-throughput assays (29–31). Furthermore, genome-wide green fluorescent protein (GFP)-tagging studies indicated that Ypl150w–GFP localized to punctate patches that appeared similar to the pattern expected for eisosome proteins (32). Consistent with this, another study found that Ypl150w bound to phosphatidylinositol lipids, including phosphatidylinositol 4,5 bisphosphate (PI_4,5_P_2_), which are enriched in the inner leaflet of the plasma membrane (33). Therefore, we examined the localization of the *C. albicans* orf19.4518 protein and found that it localized in punctate patches that partially colocalized with Sur7–mScarlet, a marker for the MCC/eisosomes in *C. albicans* (17). Other experiments indicated that the apparent plasma membrane localization may not be required for orf19.4518 function. Analysis of the phenotypes of a mutant lacking orf19.4518 showed that it is more susceptible to conditions that exacerbate cell wall stress. This indicates that orf19.4518 likely contributes to *C. albicans* virulence by promoting invasive growth and resistance to cell wall stress, which are both important for virulence. Based on these results we propose the name *CWR1* (Cell Wall Regulatory kinase) for orf19.4518.

## RESULTS

### Cwr1 protein structure

**Fig 1:**
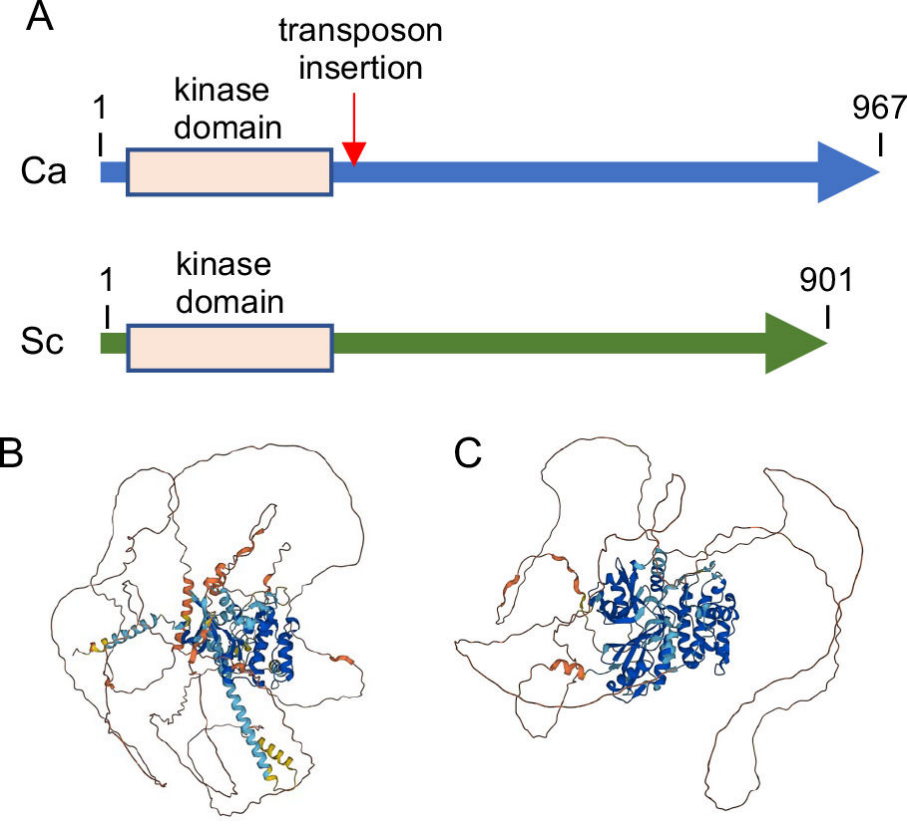
Comparison of *C. albicans* Cwr1 and *S. cerevisiae* Ypl150w proteins. (**A**) Comparison of protein length and position of the protein kinase domains in Cwr1 and Ypl150w. The arrow indicates the position of a transposon insertion in the *cwr1* mutant identified by screening the mutants described by Nobile et al. (35). (**B**) Model for the structure of Cwr1 and (**C**) Ypl150w as predicted by AlphaFold https://alphafold.com). Note that the C-terminal domains appear as disordered. A darker blue color in the ribbon diagrams indicates a very high confidence of the predicted structure (>90%), a lighter blue color indicates high confidence (70%–90%), yellow indicates low confidence (50%–70%), and orange indicates very low confidence (<50%).

The predicted Cwr1 protein is relatively large, as it is comprised of 967 amino acids. The region near the N terminus displays strong similarity to protein kinases (residues 37–283) (Fig. 1; Fig. S1). This domain contains the conserved residues that are important for protein kinase catalytic activity, as assessed by the National Library of Medicine Conserved Domains database (https://www.ncbi.nlm.nih.gov/cdd). Amino acid similarity analyzed by the Basic Local Alignment Search Tool (BLAST) indicated that the most closely related protein kinase in *C. albicans* is orf19.3751, the ortholog of *S. cerevisiae* Frk1, followed by Snf1, Gin4, and Hsl1. This indicates that Cwr1 is in the same family of yeast protein kinases that includes the calcium calmodulin-dependent protein kinases (34).

In contrast, the C-terminal region did not display similarity to any known proteins, and there were no structural predictions for this region by AlphaFold (https://alphafold.com) (36, 37) (Fig. 1B). The closest ortholog of Cwr1 in *S. cerevisiae* is a relatively uncharacterized protein kinase Ypl150w. The two proteins are similar in having a protein kinase domain at the N-terminus and a long >600 amino acid C-terminal domain (Fig. 1). Although the Cwr1 and Ypl150w protein kinase domains show approximately 50% amino acid identity, there is no significant amino acid identity in their C-terminal regions. However, the MobiDB site (https://www.mobidb.org/) predicts that the C-terminal regions of Cwr1 and Ypl150w both have characteristics similar to proteins with intrinsically disordered domains. This appears to be a conserved feature of Cwr1-related protein kinases since the human Microtubule Affinity Regulating Kinases (38), which contain protein kinase domains that are approximately 40% identical to Cwr1, also contains a long C-terminal domain with disordered character. Thus, although the C-terminal sequences are not highly conserved, they could function in a similar manner to control the function of these protein kinases since intrinsically disordered regions of protein kinases have been implicated in regulating kinase activity and mediating interaction with other proteins (39).

### Deletion of *CWR1* causes a defect in invasive growth

**Fig 2:**
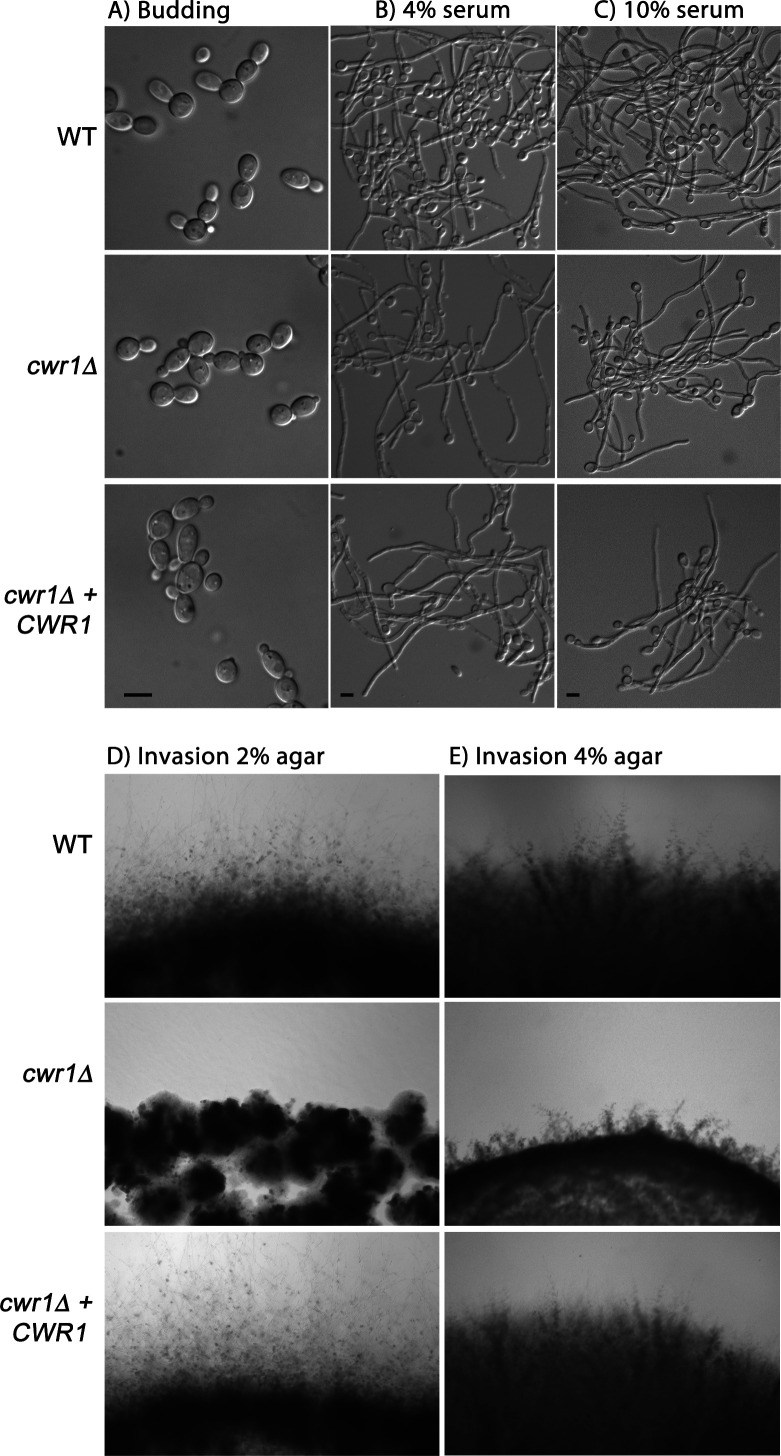
The *cwr1*Δ mutant is defective in growing invasively into agar. (**A**) Budding cells grown at 30°C in liquid synthetic dextrose medium. (**B**) Cells grown in liquid synthetic dextrose medium plus 4% bovine serum at 37°C for 2.5 h to induce hyphal formation. (**C**) Cells grown similar to those in panel (B) except that 10% bovine serum was used. Scale bars indicate 5 µm. (**D**) The indicated strains were spotted onto the surface of a 2% agar plate containing 4% bovine serum and then incubated for 2 days at 37°C. Note that the *cwr1*Δ mutant displayed limited invasive growth compared to the wild-type control and the complemented strain. (**E**) Invasive growth analysis similar to that in panel (D) except that 4% agar was used. The wild-type control strain was LLF100A, the *cwr1*Δ mutant was SNY100, and the *cwr1*Δ + *CWR1* complemented strain was SNY101. The *C. albicans* strain genotypes are described in Table 1. Similar results were observed in three independent assays.

Screening of available *C. albicans* mutant collections for strains that were defective in invasive growth (7) led to the identification of several mutants, including a strain that had a transposon insertion in orf19.4518 (*CWR1*). The mutant strain was generated by the UAU method in which transposon insertions into *C. albicans* genes were used to create homozygous mutations (35). The insertion into *CWR1* was slightly 3′ to the protein kinase domain (Fig. 1A), raising the possibility that this mutant allele might retain partial function. We therefore used CRISPR/Cas9 methods to create a homozygous mutant strain lacking the entire *CWR1* open reading frame. There were no obvious defects in budding or hyphal growth detected in liquid cultures (Fig. 2A through C). However, the *cwr1*Δ mutant strain displayed a clear defect in invasive growth into 2% agar medium containing 4% bovine serum and in more rigid medium containing 4% agar (Fig. 2D and E). The *cwr1*Δ strain could eventually begin to invade into agar after a longer incubation. Interestingly, this defect was specific to serum-induced invasive growth. There were no detectable defects in invasive growth into agar stimulated by N-acetylglucosamine, alkaline pH, or Spider medium (Fig. S2).

**TABLE 1:** *C. albicans* strains used in this study

Strain	Ref.	Short genotype	Full genotype
SN152	(40)	Parental strain	*arg4∆/arg4∆ leu2∆/leu2∆ his1∆/his1∆ URA3/ura3∆::imm434 IRO1/iro1∆::imm434*
LLF100A	(27)	Prototrophic WT control	*ARG4/arg4∆ leu2∆/leu2∆::CmLEU2 his1∆/his1∆::CdHIS1*
YLD233-1	(41)	Prototrophic WT control	*ARG4/arg4∆ leu2∆/leu2∆::CmLEU2 his1∆/his1∆::CdHIS1*
SC5314	(42)	Clinical isolate	
SNY100	This study	*cwr1Δ*	*cwr1Δ::CdARG4/cwr1Δ::CdHIS1 LEU2/leu2Δ URA3/ura3::λimm434 his1::hisG/his1::hisG arg4::hisG/arg4::hisG*
SNY101	This study	*cwr1Δ + CWR1*	*cwr1Δ::CdARG4/cwr1Δ::CdHIS1 LEU2/leu2Δ URA3/ura3::λimm434 his1::hisG/his1::hisG arg4::hisG/arg4::hisG NEUT5L::CWR1 NAT1*
SNY102	This study	*CWR1–GFP*	*CWR1–GFP::ARG4/CWR1 GFP arg4/arg4∆ leu2∆/leu2∆ his1∆/his1∆ URA3/ura3∆::imm434 IRO1/iro1∆::imm434*
SNY103	This study	*CWR1–GFP SUR7–mScarlet*	*CWR1–GFP::ARG4/CWR1–GFP::HIS1 SUR7–mScarlet::NAT1/SUR7 arg4/arg4∆ leu2∆/leu2∆ his1∆/his1∆ URA3/ura3∆::imm434 IRO1/iro1∆::imm434*
SNY104	This study	*T742–GFP SUR7–mScarlet*	*cwr1–T742–GFP::ARG4/cwr1–T742–GFP::HIS1 SUR7–mScarlet::NAT1/SUR7 arg4/arg4∆ leu2∆/leu2∆ his1∆/his1∆ URA3/ura3∆::imm434 IRO1/iro1∆::imm434*
SNY105	This study	*T527–GFP SUR7–mScarlet*	*cwr1–T527–GFP::ARG4/cwr1–T527–GFP::HIS1 SUR7–mScarlet::NAT1/SUR7 arg4/arg4∆ leu2∆/leu2∆ his1∆/his1∆ URA3/ura3∆::imm434 IRO1/iro1∆::imm434*
SNY106	This study	*T329–GFP SUR7–mScarlet*	*cwr1–T329–GFP::ARG4/cwr1–T329–GFP::HIS1 SUR7–mScarlet::NAT1/SUR7 arg4/arg4∆ leu2∆/leu2∆ his1∆/his1∆ URA3/ura3∆::imm434 IRO1/iro1∆::imm434*

### *CWR1* is important for resistance to cell wall stress

**Fig 3:**
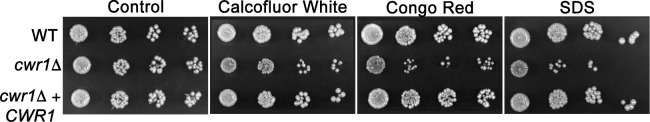
The *cwr1*Δ mutant is more susceptible to cell wall stress. A 10-fold dilution series of cells was prepared and then spotted onto synthetic complete agar medium containing no additions (control), Calcofluor White (35 µg/mL), Congo Red (35 µg/mL), or SDS (100 µg/mL). The plates were then incubated at 30°C for 2 days and then photographed to record the extent of growth inhibition for the *cwr1*Δ mutant. The *C. albicans* strains included wild-type control strain LLF100A, *cwr1*Δ mutant SNY100, and the *cwr1*Δ + CWR1-complemented strain SNY101. Similar results were observed in at least three independent assays.

Genes that promote invasive growth are often important for resistance to conditions that exacerbate cell wall stress (12, 14, 16). To examine the susceptibility to cell wall stress, a 10-fold dilution series of cells was prepared and then spotted onto the surface of agar medium containing Calcofluor White (CFW), Congo Red, or SDS, which are known to exacerbate cell wall defects. The results showed that the *cwr1*Δ mutant grew poorly under these stress conditions (Fig. 3). A control strain in which a wild-type copy of *CWR1* was introduced into the *cwr1*Δ mutant rescued these defects, indicating that the cell wall stress phenotypes were linked to the *cwr1*Δ mutation. However, the *cwr1*Δ mutant grew well under other stressful conditions including 42°C or 1 M NaCl. The latter result differs from that of a high-throughput study of *S. cerevisiae* mutants, which reported that an *S. cerevisiae* strain lacking the ortholog of *CWR1* (YPL150W) was more susceptible to osmotic stress caused by 1 M NaCl (29). Overall, these results suggest that Cwr1 promotes cell wall functions needed for both morphogenesis and stress resistance.

### The *cwr1*Δ mutant is more resistant to fluconazole

**Fig 4:**
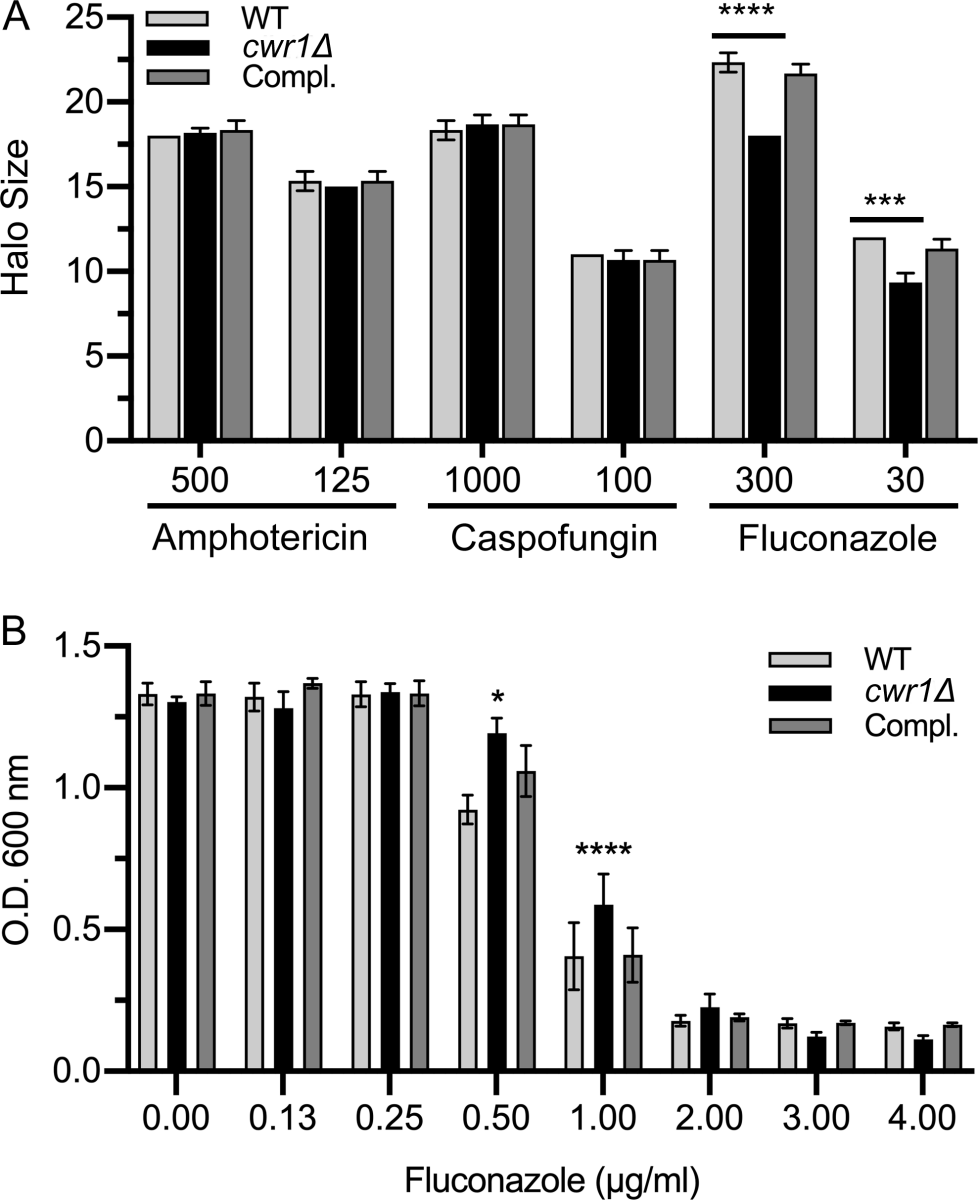
*The cwr1*Δ mutant is more resistant to fluconazole. (**A**) The wild-type control strain, the *cwr1*Δ mutant, and the *cwr1*Δ + CWR1-complemented strain were tested for susceptibility to the indicated antifungal drugs in disk diffusion assays. A lawn of the indicated cell type was spread on a complete synthetic medium agar plate, and then disks containing the indicated antifungal drug were placed on the lawn of cells. After 2 days of incubation at 30°C, the zone of growth inhibition surrounding each disk was recorded, and then the mean and SD were plotted. Ten microliters of amphotericin B, caspofungin, or fluconazole at the indicated concentration (mM) was added to the disks before they were placed on the lawn of cells. The results represent the mean and SD for three independent assays, each done in duplicate. (**B**) Cells were grown in synthetic medium in the presence of the indicated concentration of fluconazole in 96-well plates for 2 days at 37°C, and then the optical density (OD) was recorded. The results of panels (A) and (B) represent the mean and SD for three independent assays. *P < 0.05, ***P < 0.001, ****P < 0.0001 by one-way analysis of variance (ANOVA). The wild-type control strain was LLF100A, the *cwr1*Δ mutant was SNY100, and the *cwr1*Δ + *CWR1*-complemented strain was SNY101.

To determine if the *cwr1*Δ mutant displayed altered susceptibility to antifungal drugs, we assayed growth of the cells in the presence of fluconazole (FLU; inhibitor of ergosterol synthesis), amphotericin B (AmB; binds plasma membrane ergosterol), and caspofungin (inhibitor of cell wall β-glucan synthesis). Disk diffusion assays were used to quantify the effects of these drugs on the growth of the *cwr1*Δ mutant (Fig. 4A). The *cwr1*Δ mutant did not display altered response to amphotericin B or caspofungin. However, it was more resistant to fluconazole, indicated by a smaller zone of growth inhibition (Fig. 4A).

It was surprising that the *cwr1*Δ mutant was more resistant to fluconazole, since this drug inhibits the proper synthesis of sterol lipids that are important for normal plasma membrane organization and cell wall synthesis. To confirm this phenotype, the *cwr1*Δ mutant was assayed for ability to grow in liquid culture in the presence of different concentrations of fluconazole (Fig. 4B). The results confirmed that the *cwr1*Δ mutant was more resistant. It was better able to grow in the presence of fluconazole than the wild-type control strain or the *cwr1*Δ mutant complemented by reintroducing a copy of *CWR1*.

### Cwr1 protein localizes in part to eisosomes

**Fig 5:**
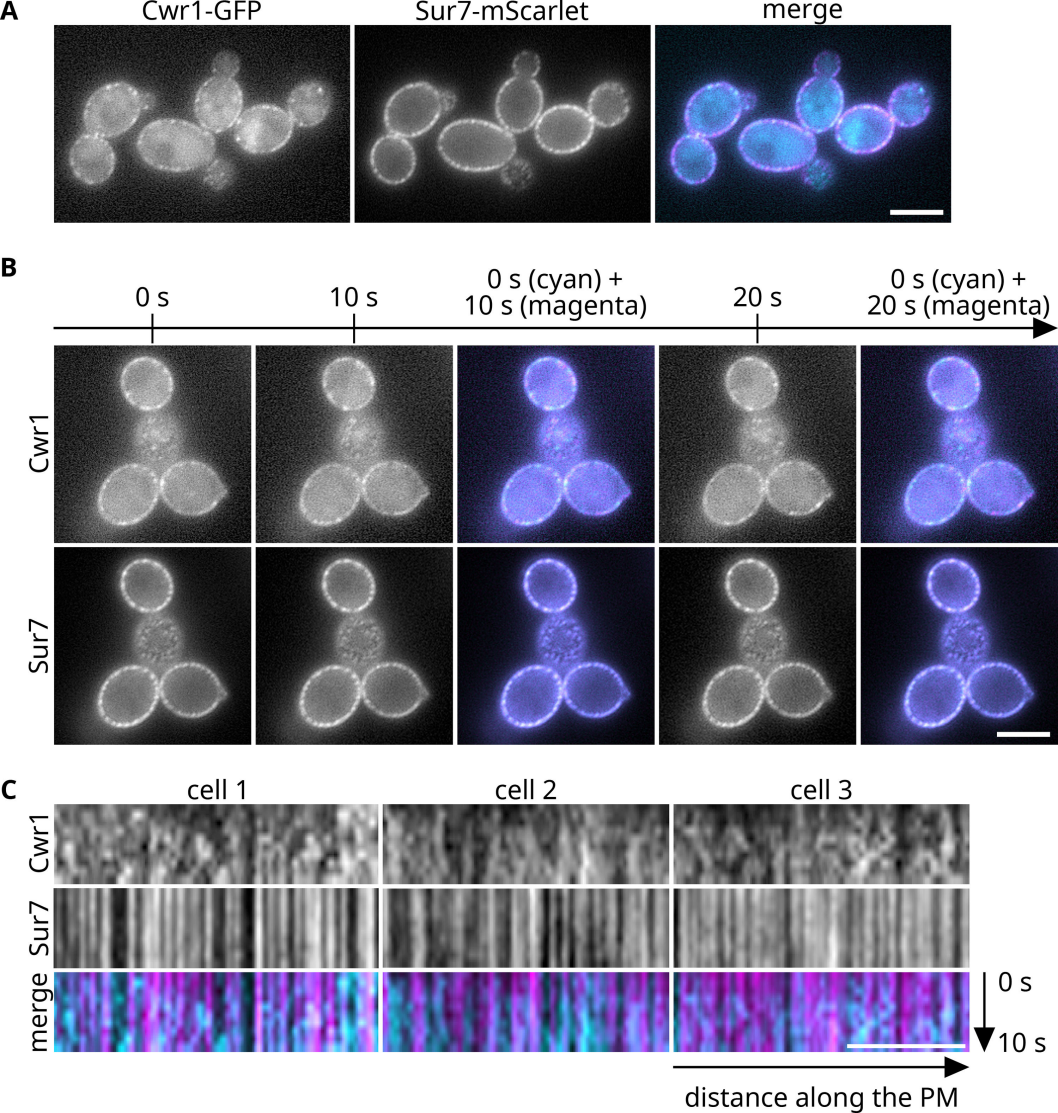
Cwr1–GFP localizes in punctate patches at the plasma membrane. (**A**) A *C. albicans* strain carrying *CWR1–GFP* and *SUR7–mScarlet* (SNY103) grown in synthetic medium was analyzed by epifluorescence microscopy. In the merged figure, Cwr1–GFP is shown in cyan and Sur7–mScarlet is shown in magenta. White patches indicate overlap. (**B**) The same cells were photographed at 10- and 20-s apart to show the mobility of the Cwr1–GFP patches, which contrasts with the stability of the Sur7–mScarlet patches. In the merged images, cyan indicates that the patch was present only at time zero; magenta indicates a new patch formed after 10 or 20 s. White patches indicate the stable patches present both at time 0 s and 10 or 20 s. (**C**) Representative kymograms were calculated from images of cells taken in 1-s intervals to show the dynamics of Cwr1–GFP localization. The horizontal and vertical arrows indicate distance along the plasma membrane (PM) and time scale, respectively. Cwr1–GFP is shown in cyan, and Sur7–mScarlet is shown in magenta. Scale bar: 5 µm.

The *S. cerevisiae* Ypl150w–GFP fusion was shown in a high-throughput study to localize in a punctate pattern at the cell periphery, suggesting it might be an eisosome protein (32). However, it was not clear whether the *C. albicans* Cwr1–GFP would localize in a similar manner since there was very limited amino acid sequence similarity outside of the protein kinase domain. The localization of *C. albicans* Cwr1 was analyzed by creating a fusion to GFP at the C-terminal end of Cwr1. The Cwr1–GFP signal observed by epi-fluorescence microscopy was very weak, so sensitivity was improved by analyzing *C. albicans* cells in which both copies of *CWR1* were fused to GFP (Fig. 5A). The results showed that Cwr1–GFP localized in a punctate pattern associated with the plasma membrane. There was also some intracellular signal that corresponded to the vacuole. GFP can build up in the vacuole because it takes time to degrade.

A variety of proteins localize in a punctate pattern (43). To determine if Cwr1 localized to MCC/eisosome domains, the cells were also tagged with a fusion between Sur7, which is known to localize to the MCC/eisosome domains, and the red fluorescent protein mScarlet. Many Cwr1–GFP patches colocalized with the eisosome patches detected by Sur7–mScarlet, indicating that Cwr1–GFP represents a novel eisosome protein. However, some Cwr1–GFP patches did not colocalize with the Sur7–mScarlet, indicating that Cwr1–GFP was able to form punctate patches at sites distinct from eisosomes. Nonetheless, this shows that Cwr1–GFP was not excluded from eisosomes as some protein are known to be (20).

A hallmark of eisosomes is that they are very stable and do not move in the plasma membrane (12, 19, 20). To examine this, cells were photographed at 10-s intervals (Fig. 5B). As expected, the Sur7–mScarlet patches did not change over the time course. In contrast, during this time frame, some Cwr1–GFP patches disappeared, and other Cwr1–GFP patches appeared at new sites, indicating that Cwr1–GFP patches are not static in the plasma membrane. To examine this further, confocal microscopy was used to image cells at 1-s intervals to create a kymogram (Fig. 5C). Whereas the Sur7–mScarlet patches appeared strictly as vertical lines in the kymogram, indicating their high stability, the Cwr1–GFP traces could be divided into groups, as vertical, disordered, and discontinuous. This indicated that both stable and mobile populations of Cwr1–GFP exist at the cell periphery.

### C-terminal sequences of Cwr1 are needed for its localization in punctate patches at the cell periphery

**Fig 6:**
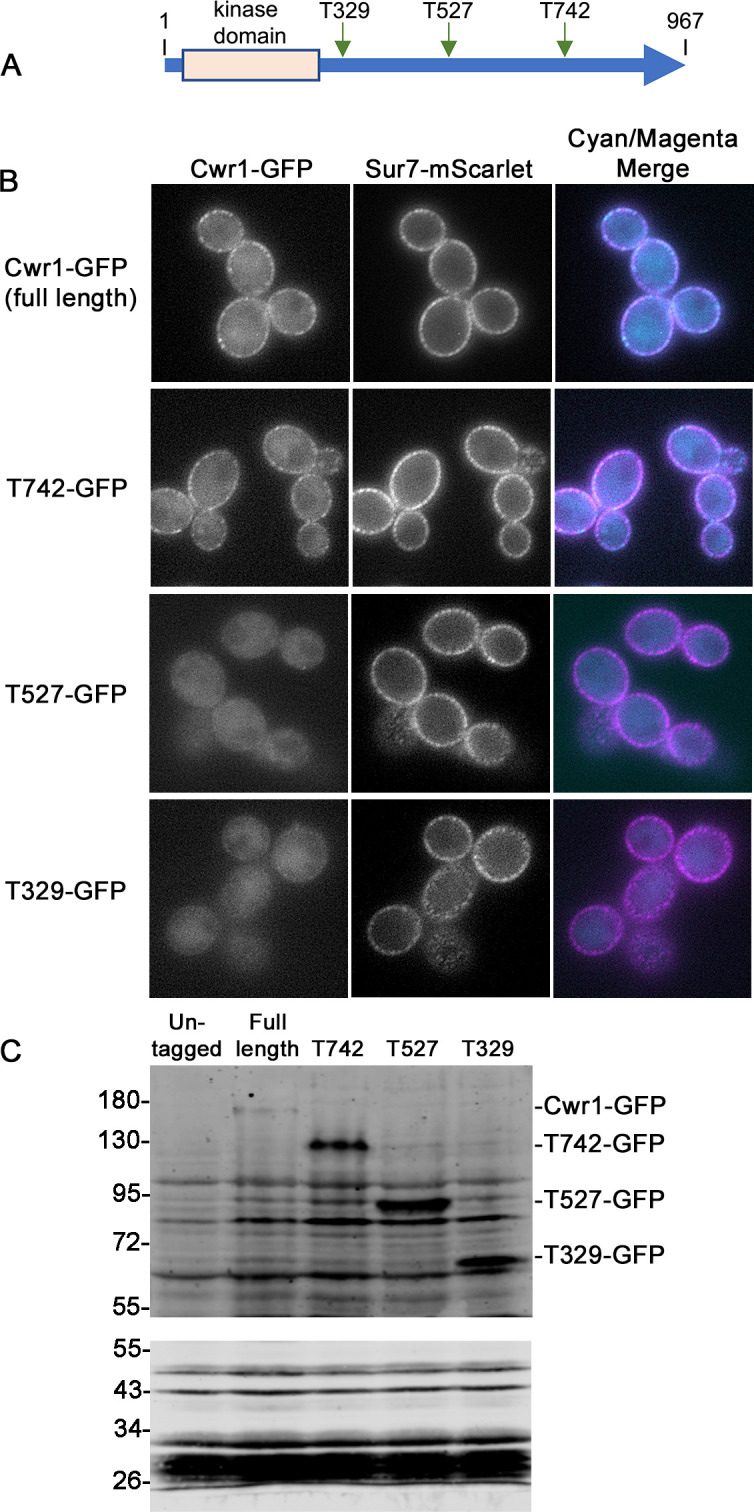
The protein kinase domain is not sufficient to localize Cwr1–GFP to punctate patches in the plasma membrane. (**A**) Truncation mutants were created by fusing GFP to the indicated sites in Cwr1. Both alleles in the genome were truncated to make the strains homozygous. (**B**) The localization of the truncated Cwr1 proteins fused to GFP was analyzed by epifluorescence microscopy in cells that lacked other *CWR1* genes. The cells also contained *SUR7–mScarlet* for comparison. (**C**) Production of Cwr1–GFP fusion proteins was analyzed on Western blots. Extracts of the indicated *CWR1–GFP* strains and a control strain that lacked GFP were probed with a mixture of two anti-GFP monoclonal antibodies. The upper blot shows extracts that were separated on an 8% polyacrylamide gel to permit better transfer of large proteins to the filter. Note that higher MW bands do not transfer well, so the full-length Cwr1–GFP detected on the blot represents an underestimate of the amount relative to the lower MW Cwr1–GFP truncation mutant proteins. The lower blot shows the same extracts that were separated on a 10% polyacrylamide gel to better resolve smaller proteins to assess whether free GFP could be detected. The *C. albicans* strains included YLD233-1, SNY103, SNY104, SNY105, and SNY106. Cells were grown in synthetic medium. Scale bar: 5 µm.

*C. albicans* Cwr1–GFP and its ortholog Ypl150w–GFP in *S. cerevisiae* both localize in punctate patches, yet they only share significant amino acid sequence similarity in their N-terminal protein kinase domains (Fig. 1). Therefore, the sequences that promote the formation of patches were investigated by inserting the GFP fusions at different sites in Cwr1 (Fig. 6A). Interestingly, fusion of GFP after codon 742 produced a protein that still localized in punctate patches (Fig. 6B). In contrast, fusion of GFP after codons 527 or 329 did not form detectable patches. Western blot analysis showed that the expected sized GFP fusions proteins were produced by the truncation mutants (Fig. 6C). This indicates that the protein kinase domain is not sufficient and that C-terminal sequences are necessary for Cwr1 to form punctate patches at the cell periphery.

### Formation of punctate patches is not essential for Cwr1 function

**Fig 7:**
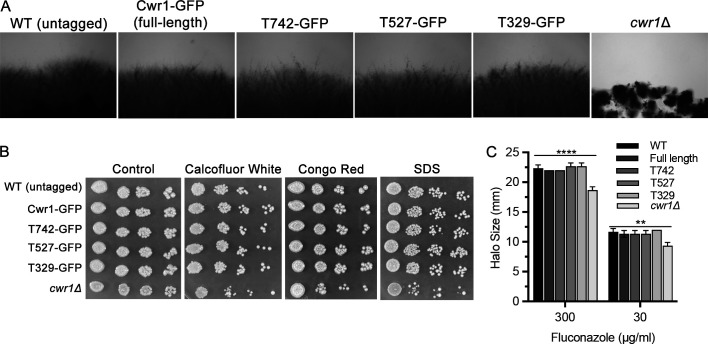
GFP-tagged *cwr1* truncation mutant strains do not display defects in invasive growth or susceptibility to cell wall stress. (**A**) The indicated strains were spotted onto the surface of a 2% agar plate containing 4% bovine serum, incubated for 2 days at 37°C, and then the edge of the spot of cells was photographed. (**B**) Aliquots of a 10-fold dilution series of cells was spotted onto synthetic complete medium agar medium containing no additions (control), Calcofluor White (35 µg/mL), Congo Red (35 µg/mL), or SDS (100 µg/mL). The plates were incubated at 30°C for 2 days and then photographed to record the extent of growth.(**C**) The indicated *C. albicans* strains were assayed for susceptibility to fluconazole using disk diffusion assays as described in Fig. 4. The X-axis indicates the concentration of fluconazole applied to the disk, and then the Y axis reports the size of the zone of growth inhibition. Note that the GFP-tagged forms of Cwr1 did not show defects in these assays relative to the wild-type control strain. The *C. albicans* strains included wild-type control strain LLF100A, SNY103, SNY104, SNY105, and SNY106. Similar results were observed in at least three independent assays.

To determine the role of the C-terminal sequences in Cwr1 function, the GFP-tagged Cwr1 mutants were analyzed for ability to undergo invasive growth. Interestingly, in contrast to the *cwr1*Δ mutant, all three truncation mutants grew invasively into 4% serum agar (Fig. 7A). Furthermore, all three truncation mutants showed wild-type levels of resistance to Calcofluor White, Congo Red, and SDS (Fig. 7B). The truncation mutants also showed wild-type levels of susceptibility to fluconazole (Fig. 7C). This indicates that the formation of punctate patches associated with the plasma membrane is not essential for Cwr1 function. One possibility is that the patches represent inactive forms of Cwr1, so it is not critical for them to form for Cwr1 to function.

### Localization of Cwr1–GFP to eisosomes under stress conditions

**Fig 8:**
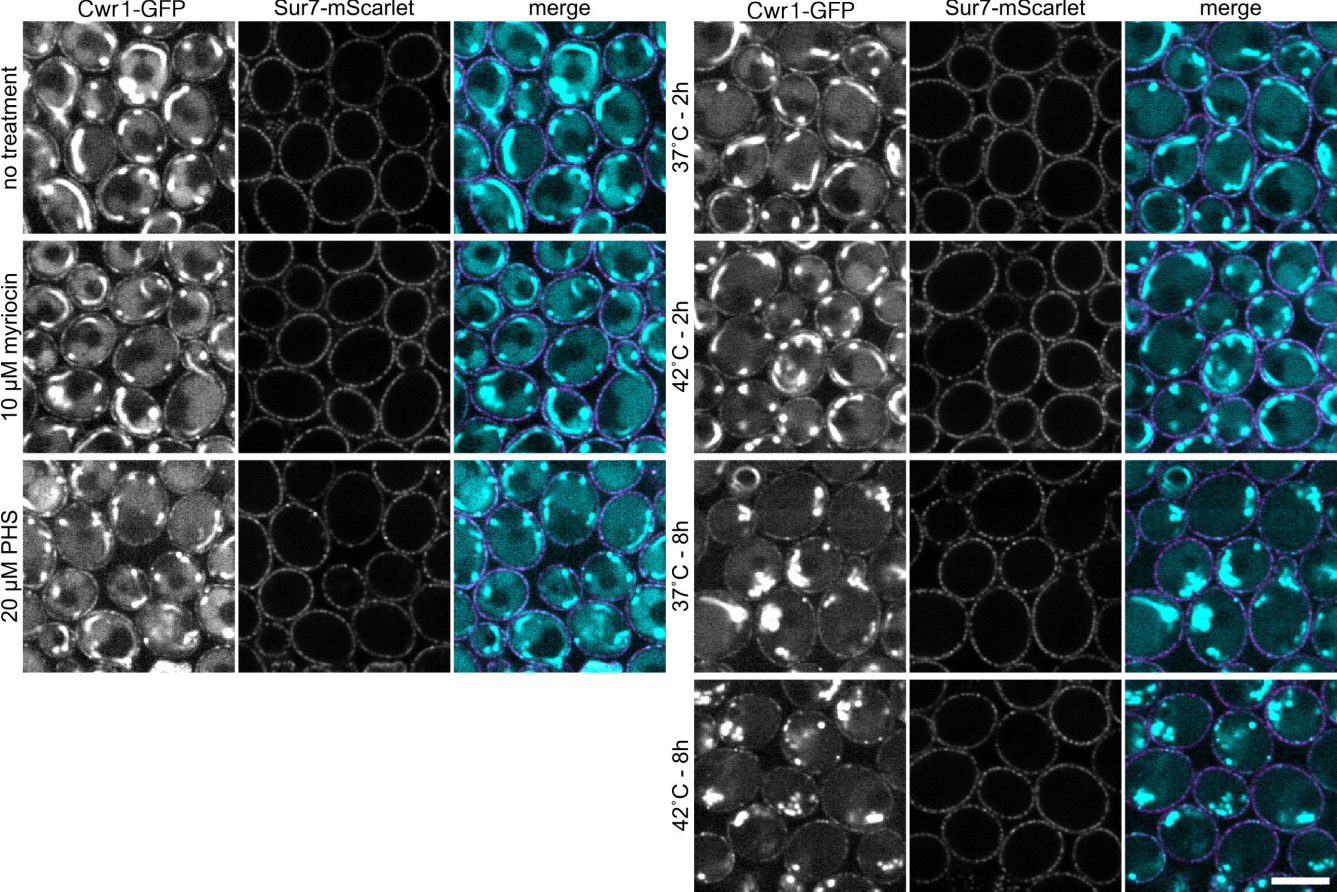
Analysis of Cwr1–GFP in response to stress. A *C. albicans* strain carrying *CWR1–GFP* and *SUR7–mScarlet* was grown at 30°C for 6 h without treatment/stress and then grown for two additional hours in the presence of 10 µM myriocin, 20 µM phytosphingosine (PHS), or after being shifted to 37°C or 42°C. Alternatively, the cells were grown at 37°C or 42°C for 8 h, as indicated. The cells were then analyzed by confocal microscopy, where 10 consecutive images with 1-s acquisition time were recorded and the signal summed after drift and bleaching correction. Cells were grown in synthetic medium. Scale bar: 5 µm.

**Fig 9:**
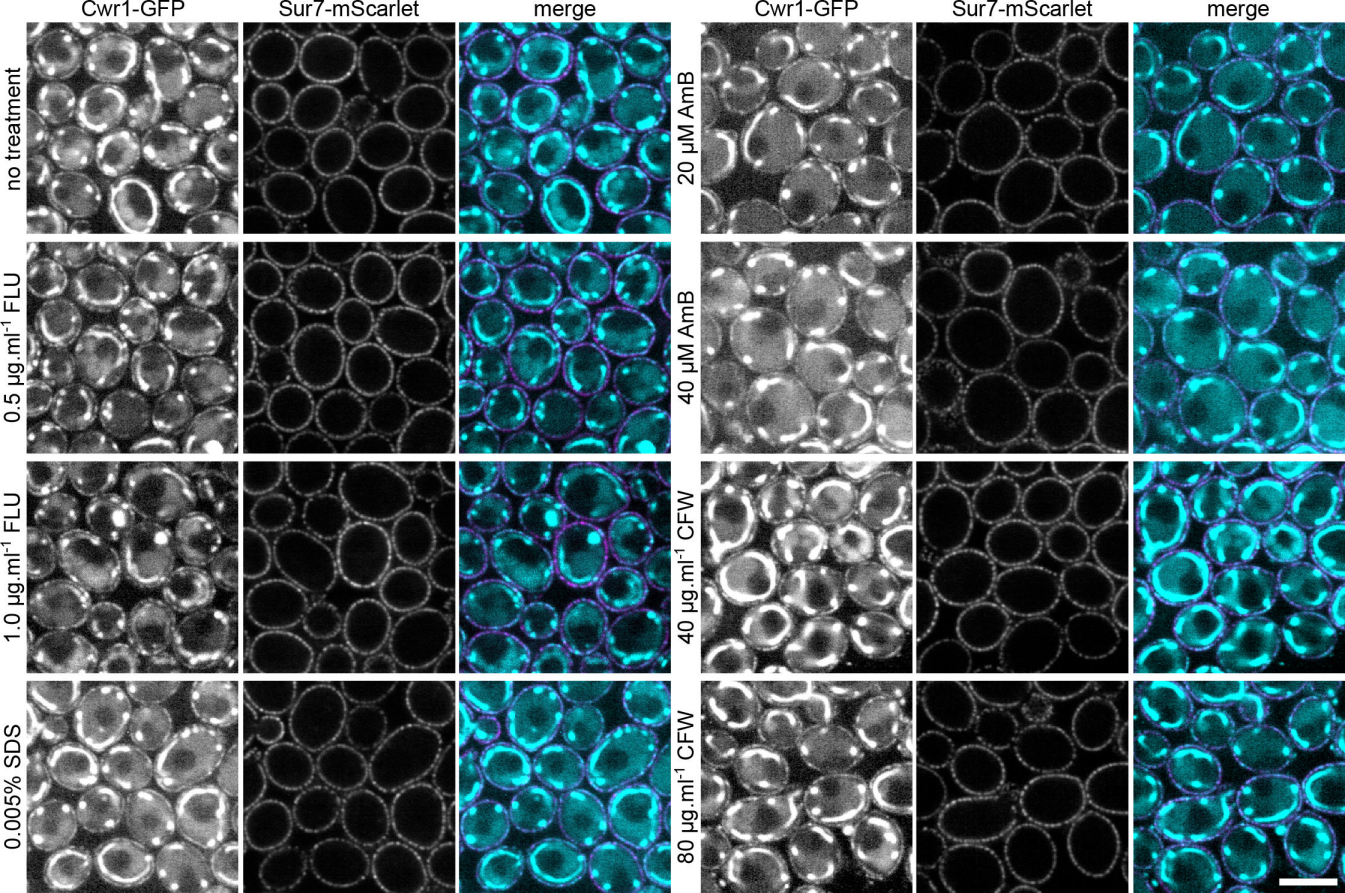
Analysis of Cwr1–GFP in response to antifungal drugs and cell wall stress. *C. albicans* cells carrying *CWR1–GFP* and *SUR7–mScarlet* were grown at 30˚C for 6 h without treatment and then for 2 h in the presence of the indicated concentration of fluconazole, SDS, amphotericin B (AmB), or Calcofluor White (CFW). The cells were then analyzed by confocal microscopy, where 10 consecutive images with 1-s acquisition time were recorded and the signal summed after drift and bleaching correction. Cells were grown in synthetic medium. Scale bar: 5 µm.

Previous studies have shown that some proteins enter or leave eisosomes under different conditions. For example, Nce102 and Slm1/2 leave after inhibition of sphingolipid synthesis (22, 26), whereas Xrn1 localizes to eisosomes after the post-diauxic shift in yeast growth (44). Therefore, the localization of Cwr1–GFP was analyzed under different stress conditions using a spinning disc confocal microscope. Spinning disc confocal microscopy resulted in a much higher level of detection of cytoplasmic fluorescence in the Cwr1–GFP cells (Fig. 8 and 9). Subsequent studies revealed that the synthetic medium used in these studies caused the cytoplasmic fluorescence (Fig. S3). However, the patchy distribution of Cwr1–GFP at the plasma membrane was still readily detectable.

The localization of Cwr1–GFP was analyzed after membrane stress caused by inhibition of sphingolipid synthesis with myriocin and by increasing the level of sphingolipids by addition of phytosphingosine (Fig. 8). The localization of Cwr1–GFP was also analyzed after growth at 42°C, which is stressful to cells. The results indicate that there was no major change in the pattern of Cwr1–GFP localization (Fig. 8). Quantitative analysis also failed to show a difference in the number of Cwr1–GFP patches or their intensity (Fig. S4).

The localization of Cwr1–GFP was then analyzed under stress due to the antifungal drugs fluconazole and amphotericin B, and after treatment with stressors that affect the plasma membrane (SDS) and the cell wall (Calcofluor White) (Fig. 9). However, these conditions also did not appear to have a major effect on the localization of Cwr1–GFP (Fig. 9; Fig. S4). Thus, in contrast to some other eisosome proteins, the localization of Cwr1–GFP was not detectably altered in response to a range of different stressful conditions. This suggests that other mechanisms may influence Cwr1 function. For example, Cwr1 may differentially act on other proteins that enter or leave eisosomes in response to stress.

## DISCUSSION

The ability of *C. albicans* to undergo invasive hyphal growth promotes virulence, especially in the case of oral infections (7, 9, 10, 45, 46). To better define the genes that regulate these processes, we screened libraries of *C. albicans* mutants to identify genes that promote invasive hyphal growth (7). This led to the identification of a poorly studied protein kinase (orf19.4518) we have named Cwr1 (Cell Wall Regulatory kinase). Interestingly, the *cwr1*Δ invasive growth defect was observed most obviously in agar medium containing bovine serum as the inducer (Fig. 2). Other conditions such as GlcNAc, alkaline pH and Spider medium were able to induce invasive growth of the *cwr1*Δ strain (Fig. S2). This was surprising since serum is one of the stronger inducers of invasive growth into agar. Also, it was not obvious that the *cwr1*Δ mutant had a defect in inducing hyphae in liquid culture in response to serum (Fig. 2). Thus, Cwr1 appears to play a specific role in promoting invasive growth in response to serum. This phenotype may be related to a partial defect in biofilm formation reported for a *cwr1*Δ mutant that was identified by screening a collection of *C. albicans* protein kinase mutants for the ability to form biofilms (47).

Many mutant *C. albicans* strains that are defective in invasive hyphal growth are also defective in resisting stress caused by the perturbation of the plasma membrane or cell wall. This was true of the *cwr1*Δ mutant as it was more susceptible to growth inhibition by Calcofluor White, Congo Red, and SDS (Fig. 3). Consistent with this, a separate analysis of a set of *C. albicans* protein kinase mutants showed that *cwr1*Δ cells were more susceptible to killing by chitosan, a deacetylated form of chitin that has antimicrobial properties (48). Fifteen out of the 21 chitosan-sensitive mutants that were identified were known to be altered in cell wall or plasma membrane integrity, further strengthening the evidence for a role of Cwr1 in stress resistance. Interestingly, an analysis of signaling networks in *S. cerevisiae* suggested that the Cwr1 ortholog (Ypl150w) acts in a network of other protein kinases that includes the stress-responsive protein kinase Hog1 (49). A different high-throughput study in *S. cerevisiae* indicated that YPL150W was needed for resistance to 1 M NaCl, but increased sensitivity to osmotic stress was not seen for the *C. albicans cwr1*Δ mutant. Thus, although both Cwr1 and Ypl150w have roles in stress response, their specific functional roles may be distinct. Consistent with this, only the protein kinase domains share significant amino acid sequence conservation between these two orthologs, whereas the long C-terminal domains are distinct (Fig. 1). In contrast to the increased susceptibility to agents that cause cell wall stress, it was unexpected that the *cwr1*Δ mutant was more resistant to fluconazole (Fig. 4), which is known to cause plasma membrane stress by altering ergosterol production. One possibility is that the altered cell wall upregulates stress-responsive pathways that make the *cwr1*Δ mutant more resistant to fluconazole.

A high-throughput study of GFP-tagged proteins in *S. cerevisiae* detected Ypl150w–GFP in punctate patches at the periphery of the cell, suggesting it could be a component of eisosome domains in the plasma membrane (32). Eisosomes contain a diverse set of proteins, many of which are involved in the response to stress and regulation of morphogenesis (12). Therefore, we analyzed Cwr1–GFP and showed that it could partially colocalize with Sur7–mScarlet, a known component of MCC/eisosomes (Fig. 5). However, the Cwr1–GFP patches associated with the plasma membrane were distinct in that they were very dynamic, which contrasts with the stable localization Sur7–mScarlet to eisosomes.

The C-terminal sequences of Cwr1 were needed for its localization to punctate patches associated with the plasma membrane. Fusing GFP after the full-length 967-amino acid protein or after amino acid 742 resulted in proteins that localized to the membrane-associated patches, whereas Cwr1 proteins fused to GFP after amino acids 527 or 329 did not. Interestingly, all of these GFP fusion proteins were able to promote invasive growth and stress resistance, whether or not they localized to the membrane-associated patches (Fig. 7). Although this suggests that the punctate localization of Cwr1–GFP is not critical for its function, there could be advantages under some conditions. The potential role of Cwr1–GFP localization was analyzed further by exposing cells to different kinds of stress conditions that affect the plasma membrane or cell wall. However, none of these conditions strongly impacted the localization of Cwr1–GFP (Fig. 8 and 9). Thus, in contrast to some other eisosome proteins that move in or out of eisosomes in response to stress (12, 26, 44, 50), the degree of colocalization between Cwr1–GFP with eisosomes did not appear to change in response to stress. Although it is unclear why Cwr1–GFP clusters in punctate patches, it is interesting that many other proteins that play key regulatory roles are found in similar types of punctate patches associated with the plasma membrane. For example, the Tor2 complex, the pH-sensing complex, the Mss4 lipid kinase that is needed to create phosphatidylinositol 4,5 bisphosphate, and sensors of osmolarity and cell wall integrity all form punctate patches associated with the plasma membrane (43, 51–56). Since the truncated versions of Cwr1 remained functional without forming punctate patches (Fig. 7), this could suggest that protein aggregation may play a regulatory role that is not essential for function. Perhaps disassembly of Cwr1 patches could lead to mobilization of a previously inactive pool of the protein. In this regard, it is interesting that some nutrient transporters leave the MCC compartment associated with eisosomes when they become active (57, 58), and Xrn1 is released from eisosomes into the cytosol when activated by the supply of fermentable sugar (59).

The level of Cwr1–GFP stayed relatively similar under the different stress conditions, with a slight increase detected after treatment with amphotericin B (Fig. S4). Consistent with this, there were no major changes in *CWR1* mRNA levels under different types of stress conditions including changes in pH, cell wall stress, oxidative stress, and nitrosative stress (60). This is not surprising, since protein kinases are not typically regulated transcriptionally, but are instead usually regulated by accessory proteins or changing conditions in the cell.

Altogether, these results identify Cwr1 as a protein kinase that forms mobile punctate patches associated with the plasma membrane that can overlap with eisosomes. Analysis of the *cwr1*Δ mutant indicates that Cwr1 promotes invasive hyphal growth and resistance to stress. A better understanding of these processes is needed to aid in the development of novel therapeutic approaches (12, 61). This indicates that Cwr1 contributes to functions that can promote the virulence of *C. albicans*.

## MATERIALS AND METHODS

### Strains and media

The *C. albicans* strains are described in Table 1. Cells were grown in rich YPD (yeast extract, peptone, dextrose) medium or complete synthetic medium containing Yeast Nitrogen Base, dextrose, amino acids, and uridine (62). Libraries of *C. albicans* mutant strains (35, 63, 64) were obtained from the Fungal Genetics Stock Center (65).

The *C. albicans cwr1*Δ mutant was constructed by deleting both copies of the *CWR1* gene from *C. albicans* strain SN152 (40). The oligonucleotide primers are listed in Table S1. PCR was used to create deletion cassettes using as a template plasmid pSN52 (*C. dubliniensis HIS1*) or pSN69 (*C. dubliniensis ARG4*) (40). The primers contained approximately 80 bp of homology to either the 5′ or 3′ end of the *CWR1* open reading frame to target the deletion cassette. PCR was conducted with Ex Taq polymerase (TaKaRa Bio, Inc.). DNA was introduced into cells by electroporation. Deletion mutants were identified by PCR amplification of genomic DNA using primers that flanked the 5′ and 3′ ends of the genes as well as internal primers. Six independent *cwr1*Δ isolates were examined to verify that they displayed the same phenotype. A complemented strain in which a copy of *CWR1* was reintroduced into the *cwr1*Δ mutant strain was created by first using PCR to amplify the wild-type *CWR1* gene along with 500 bp upstream and 350 bp downstream sequences. The primers also contained 80 bp of homology to the ends of *Sma* I-digested pDIS3. The *CWR1* gene was then inserted into *Sma* I-cleaved pDIS3 by gap repair in *S. cerevisiae* strain W3031A (66). The resulting pDIS3–*CWR1* plasmid was then digested with *Sfi* I to release a cassette containing *CWR1* and a *NAT1* selectable marker flanked by sequences corresponding to the NEUT5L locus. This DNA fragment was introduced into the *cwr1Δ* mutant to integrate a copy of *CWR1* at the neutral locus NEUT5L.

GFP-tagged strains were created using a more photostable version of GFP called GFPγ that was codon optimized for *C. albicans* (67). To enhance the weak signal, GFPγ was fused to the C-terminal coding sequence of both alleles of *CWR1*. Fusion of GFP to one allele was selected for by *ARG4*, and the fusion to the other allele was selected for with *HIS1*. The *CWR1–GFP* fusions were created in a strain that also contained *SUR7–mScarlet* (68) to facilitate comparison of Cwr1–GFP localization with eisosomes. Truncated versions of *CWR1–GFP* were created in a similar manner by inserting the GFP cassette after the indicated codon. We confirmed the production of the expected Cwr1–GFP fusion proteins by Western blotting. *C. albicans* strains were grown overnight in YPD at 30°C with shaking until a density of ~1 × 10^7^ cells/mL. Cells were harvested by centrifugation and then lysed in Laemmli buffer (2% SDS, 10% glycerol, 125 mM Tris HCl, pH 6.8, and 0.002% bromophenol blue) by agitation with zirconia beads. 2-Mercaptoethanol was added to 5% final concentration, and then the samples were boiled at 100°C for 10 min. Proteins were resolved by SDS-PAGE and transferred to a nitrocellulose membrane using a semi-dry transfer apparatus. Blots were probed with a mix of two anti-GFP monoclonal antibodies (Takara Bio Inc. cat. # 632381JL-8 and cat. # 632569 EGFP) at a 1:1,000 dilution in TBS-T (0.1% Tween-20, 2% [wt/vol] bovine serum albumin, and 0.2% [wt/vol] sodium azide) buffer for 12 h. The blots were washed and then incubated with anti-rabbit IgG secondary antibody (IRDye 800-conjugated, LI-COR Biosciences, Lincoln, NE) diluted 1:10,000 in TBS containing 0.3% Tween-20. Blots were scanned with an Odyssey CLx Infrared Imaging System (LI-COR Biosciences), and the images were analyzed using ImageStudio software (LI-COR Biosciences).

### Agar invasion assays

As described previously (7), libraries of deletion mutant strains (39, 60, 61), along with strains from our lab. were screened for invasive growth into 1.5% agar containing 4% fetal bovine serum. A *cwr1* transposon insertion mutant was identified as being partially defective, and then PCR was used to confirm that the expected gene was deleted in the mutant strain. The *cwr1*Δ complete deletion mutant was then used in the subsequent studies where it was tested for invasive growth under a broader range of conditions including GlcNAc (Yeast Nitrogen base with 50 mM GlcNAc), alkaline pH 8 (150 mM BICINE pH 8 buffer), and spider medium (Yeast Nitrogen Base, 1% mannitol, 1% nutrient broth, 0.2% K_2_HPO_4_, pH 7.2 before autoclaving). Plates were incubated at 37°C.

### Susceptibility to cell wall stress

The ability of cells to grow under conditions that exacerbate cell wall defects was tested by growing cells on agar medium plates at high temperature (42°C) or in the presence of Calcofluor White, Congo Red, and SDS. The cells were grown overnight, and then 10-fold dilution series of each strain was prepared. A micropipette was used to spot 3 µl of each dilution on the surface of YPD plates containing the following stress agent Calcofluor White (35 µg/mL), Congo Red (35 µg/mL), or SDS (100 µg/mL). The agar plates were incubated at 30°C, except for those that were incubated at 42°C. The plates were photographed after 2 days to record the relative amounts of growth under each condition.

### Susceptibility to antifungal drugs

Susceptibility to antifungal drugs was tested using disk diffusion assays. Cells from a fresh overnight culture were harvested by centrifugation, resuspended at 1.0 × 10^6^ cells/mL, and then 250 µL was spread onto the surface of a synthetic medium agar plate. Filter disks carrying the indicated concentration of antifungal drugs were placed on the lawn of cells, the plates were incubated at 30°C for 2 days, and then the sizes of the zones of growth inhibition surrounding each filter disk were measured. The results represent the average of three independent results, each done in duplicate. The differences in the size of the zones of growth inhibition were assessed for statistical significance by analysis of variance (ANOVA) with a Tukey’s multiple comparison test using GraphPad Prism after assessment of equal variance (Brown–Forsythe) and normality (Shapiro–Wilk).

To confirm the increased resistance of the *cwr1*Δ mutant to fluconazole, the wild-type control and mutant strains were grown in 96-well plates in synthetic medium containing a twofold dilution series of fluconazole. The plates were incubated at 37°C for 2 days. The extent of cell growth was then assayed in a 96-well plate reader. The results represent the average of three independent assays, each done in duplicate. The statistical significance of the differences in the extent of growth in the presence of fluconazole was determined by ANOVA with a Tukey’s multiple comparison test using GraphPad Prism after assessment of equal variance (Brown–Forsythe) and normality (Shapiro–Wilk).

### Fluorescence microscopy

Epi-fluorescence microscopy of Cwr1–GFP strain was carried out using a Zeiss Axio Observer 7 microscope. Cells were grown in synthetic medium with dextrose to log phase and then harvested. Images were recorded using a Zeiss AxioCam 702 digital camera and then processed using Zeiss ZEN software.

Confocal microscopy was used to assess the effects of stress on the cellular distribution of Cwr1–GFP. Cells grown overnight at 30°C in synthetic complete medium were diluted into fresh medium and grown for 6 h, unless specified otherwise. Stress was induced by addition of the following agents: amphotericin B (AmB, 5 mM DMSO stock; Sigma, A2411), Calcofluor White (CFW, 10 mM water stock; Sigma, F3543), fluconazole (1 mg/mL ethanol stock; Sigma, F8929), myriocin (MYR, 2 mg/mL methanol stock; Sigma, M1177), phytosphingosine (PHS, 5 mg/mL ethanol stock; Sigma, P2795), or sodium dodecyl sulfate (10% water stock; Sigma, 436143) to the indicated concentration, and then incubated for an additional 2 h. Alternatively, cultures grown at 30°C for 6 h were shifted to either 37°C or 42°C for 2 or 8 h.

Cells were concentrated by centrifugation and then 1 µL of cell suspension was immobilized on a 0.17-mm cover glass by a thin film of 1% agarose prepared in 50 mM potassium phosphate buffer (pH 6.3). The cells were imaged using an Olympus SpinSR10 confocal spinning disc microscope with super-resolution mode, equipped with a ×60 Extended PlanApochromat (UPLXAPO) oil immersion objective (numerical aperture [NA], 1.42). GFP and mScarlet were excited with solid state 488- and 561-nm lasers using respective excitation filters, and the signal was acquired with 2× Hamamatsu ORCA-Flash 4.0 CMOS camera for two-channel imaging. Due to the low Cwr1–GFP signal, 10 consecutive images with 1-s acquisition time were recorded and the signal summed after drift and bleaching correction.

### Confocal microscope image analysis

Image processing and analysis of confocal microscope images was performed in Fiji (ImageJ 1.54 f) using custom-written macros used previously (69–71) and updated for the purpose of this study. These macros are available at https://github.com/jakubzahumensky/microscopy_analysis. We recently published a detailed description of their use (72).

Cell segmentation masks used for the analysis were made using Cellpose software (73). In brief, the raw microscopy images were drift corrected and cropped to exclude zero-intensity areas, corrected for bleaching ("Histogram Matching" method), and summed. These were used to make cell segmentation masks in Cellpose with parameters set in a way that the mask edges intersect plasma membrane patches in their middles. Incompletely imaged cells were removed automatically at this step. The segmentation masks were converted into regions of interest (ROIs) in Fiji, fitted with ellipses as approximations of cells, and curated manually to remove or adjust incorrectly created ROIs and remove ROIs of actively budding cells. For each cell, multiple parameters were automatically quantified, including mean and integrated fluorescence signal intensity (in the whole cell, plasma membrane, and cell interior), cell cross-section area, and the number of high-intensity patches in the plasma membrane. The mean values of parameters of interest were calculated from all cells within each biological replicate and respective condition. From these, the final mean and standard deviation were calculated and plotted.

Due to the low signal-to-noise ratio of Cwr1–GFP, we were unable to use the traditional Pearson’s, Mander’s, and Spearman’s coefficients to analyze the colocalization of Sur7 and Cwr1. We circumvented this issue as follows: each channel of the raw images was segmented using the Graylevel Watershed Fiji plugin (developed by the Biomedical Imaging Group at EPFL: https://bigwww.epfl.ch/sage/soft/watershed/), resulting in binary images. These were then filtered using a binary mask obtained from the regions of interest (ROIs; corresponding to cells to be analyzed) for respective images, resulting in binary images (one for each channel) of small objects whose positions corresponded to the high-intensity plasma membrane patches. These were used to count the number of patches per cell – Fig. S3A and B. Subsequently, the binary images for the two images were multiplied, which gave us a binary image of their overlap. After removing small objects (using the “Despeckle” plugin in Fiji), the number of objects was counted. To quantify the overlap of Cwr1 and Sur7 patches, the number of objects in the overlap binary image was divided by the number of patches in the image of the respective Cwr1/Sur7 channel (Fig. S4C and D).

The statistical significance of differences between control and individual conditions was assessed by performing individual Student’s *t*-tests in SigmaPlot 15 (Systat). All analyzed pairs passed both normality (Shapiro–Wilk) and equal variance (Brown–Forsythe) tests before *P*-values were calculated.

Note on normalization: Mean cell intensities were normalized to controls as follows. The average across conditions within a biological replicate set was calculated, and the mean of each condition was divided by this value. Subsequently, the average value of the obtained means for controls across biological replicates was calculated. All individual values across all replicates and conditions were then divided by this value. This way, the control is set to 1 and retains a standard deviation.

## SUPPLEMENTAL MATERIAL

Figure S1 msphere.00391-24-s0001.pdf Multiple sequence alignment of Cwr1 with orthologs from *S. cerevisiae* and *H. sapiens*.

Figure S2 msphere.00391-24-s0002.pdf The *cwr1*Δ mutant grows invasively into agar containing alkaline pH, GlcNAc, or Spider medium.

Figure S3 msphere.00391-24-s0003.pdf Comparison of Cwr1-GFP in cells grown in different media.

Figure S4 msphere.00391-24-s0004.pdf Quantitative analysis of Cwr1-GFP localization.

Table S1 msphere.00391-24-s0005.pdf List of oligonucleotides used as primers to construct mutant strains and strains tagged with fluorescent proteins.

ASM does not own the copyrights to Supplemental Material that may be linked to, or accessed through, an article. The authors have granted ASM a non-exclusive, world-wide license to publish the Supplemental Material files. Please contact the corresponding author directly for reuse.
