# Supplementary material for: The Cwr1 protein kinase localizes to the plasma membrane and mediates resistance to cell wall stress in Candida albicans
Source: mSphere. 2024 Nov 29;9(12):e00391-24. doi: 10.1128/msphere.00391-24 (PMC11656795; doi:10.1128/msphere.00391-24)
Supplement: Figure S1 — Multiple sequence alignment of Cwr1 with orthologs from S. cerevisiae and H. sapiens. [file msphere.00391-24-s0001.pdf]

```
HsMARK1     MSARTPLPTVNERDTENHTSVDGYTEPHIQPTKSSSRQNIPRCRNSITSATDEQPHIGNY     60
CaElk1      --------------------MNGIGSSKVINN--TAQLAAQFNDFYLEITSPKISQIGNY     38
ScYpl150w   -------------------MVNPVGSSKLEQNNIKSIIGSSYNRLYSQFTSDELTEVGNY     41
                                ::   . ::  .  .:               :: :  .:***

HsMARK1     RLQKTIGKGNFAKVKLARHVLTGREVAVKIIDKTQLNPTSLQKLFREVRIMKILNHPNIV     120
CaElk1      KIIKEIGEGAFGKAYLATHILLNINVVLKCGLIDD------PNIVREIYYHKQLKHKNIV     92
ScYpl150w   KILKQIGEGSFGKVYLALHRPTHRKVCLKTSDKND------PNIVREVFYHRQFDFPYIT     95
            :: * **:* *.*. ** *       :* :*       :         ::.**:   : :..  *.

HsMARK1     KLFEVIETEKTLYLVMEYASGGEVFDYLVAHGRMKEKEARAKFRQIVSAVQYCHQKYIVH     180
CaElk1      SLYEVIKTENHLWIALEYCQGGELYYYIYEKKRLELDECRNIFFQIVLGVKYVHSLNLSH     152
ScYpl150w   KLYEVIVTESKVWMALEYCPGKELYDHLLSLRRISLLECGELFAQISGAVYYAHSMHCVH     155
            .*:*** **. :::.:**. * *:: ::    *:.   *.    * **  .* * *.    *

HsMARK1     RDLKAENLLLDGD--MNIKIADFGFSNEFTV--GNKLDTFCGSPPYAAPELFQGKKYDGP     236
CaElk1      RDLKLENILLADQKRTIVKLTDFGFIREFNPQSRKFLSTICGTTVYMAPELLTGQKYSGF     212
ScYpl150w   RDLKLENILLDKN--GNAKLTDFGFTRECMT--KTTLETVCGTTVYMAPELIERRTYDGF     211
            **** **:**  :      *::**** .*       . *.*.**:  * ****:  :.*.*

HsMARK1     EVDVWSLGVILYTLVSGSLPFDGQNLKELRERVLRGKYRI-PFYMSTDCENLLKKLLVLN     295
CaElk1      VIDIWSMGVILYTMLNGMLPFDDDDEMKIQHKVINTEPMFYDH-VPIDVNQLISKMLSKD     271
ScYpl150w   KIDIWSLGVILYTLITGYLPFDDDDEAKTKWKIVNEEPKYDAKVIPDDARDLISRLLAKN     271
             :*:**:******::.* ****.::  : : :::. :           :  * .:*:.::*  :

HsMARK1     PIKRGSLEQIMKDRWMNVGHEEE-------------------ELKPYTEPDPDFNDT--     333
CaElk1      PNQRPSLNEILNSSYLIDVYNKYLEKGTKRNSSGSGDAESIISINQHYNTVDRPFEAKIE     331
ScYpl150w   PGERPSLSQVLRHPFLQPYGSVVLDQTQK-------------ILCRQRSGGTQFKSKLE     317
             * :* **.:::.  ::     .                          .  *: .

HsMARK1     -KRIDIMVTMGFARDEINDALINQKYDEVMATYILLGRKPPEFEGG-------------     378
CaElk1      KDLLRKLQRISFDTEELKATMYNNEINSLTAFYELLLTQEYSKKKQKYMREKKRKLYEAK     391
ScYpl150w   RRLLKRLKQSGVDTQAIKQSILKKKCDSLSGLWLLLLAQGKKQENCKYPKRSRSVL-SVK     376
                :  :   ..  : :: :: ::: :.: . : **  :  . :

HsMARK1     ESLSSGNL-------CQRSRPSSDLNNSTLQSPAHLKVQR--SISANQKQ----------     419
CaElk1      KSLKKSRK---------RVKSVLSLSDQASGSQPLERIMSSLSIASNKNSSRHTS--TIA     440
ScYpl150w   KVIESATHNDTNGISEDVLKPSLELSRAASLS----K-------MLNKGSDFVTSMTPVS     425
            : :...                :   .*.  :  *    :          *: .

HsMARK1     RR-FSDHAGPSIPPAVSYTKRPQANSVESEQKEEWDKDVA---------RKLGSTTVGSK     469
CaElk1      RKSLDKNDTTQIPPSPKSSQTSRLNIEIP-SPRATNTSIALPPRSRRGSREISTTTAAS-     498
ScYpl150w   RKKS-KDSAKVLNPT---------LSKIS-SQRAYSHSIAGSPRKSNN------------     462
            *:   ..   : *:               . .  . .:*

HsMARK1     SEMTASPLVGPERKKSSTIPSNNVY-------------------SGGSMARRNTYVCER     509
CaElk1      -S-ITADLNTPLRRTVSFVPDGRRL----SQVISSEPKETIKKTNKSGKILHKLQFWKKN     552
ScYpl150w   ----------FLQKVSSFFKSKKSSNSNSNNSIHTNVSESLIASNRG--APSSGSFLKKN     510
                      :: * . . .                         .       :  :.

HsMARK1     TTDRYVAL------QNGKDSSLTEMSVSSISSAGSSVASAVP-SARP-RHQKSMSTSGHP     561
CaElk1      KGDEYQD--DTISHYSTKSNHSKSTNDVMIESDGENPLELIV-KGGNNSPIKFDNTMNSH     609
ScYpl150w   SGSIQKSRTDTVANPS------R---TESIGSLNENVAGAIVPRSANNTTLENKKTSGNE     561
            . .           .            * * ...    :    .        :  .* .

HsMARK1     IKVTLPTIKDGSEAYRPGSTTQRVPAASPSAHSISTATPDRTRFPRGSSSRS------TF     615
CaElk1      QKM-----------------NTVPPEVL-------EQQRLDRFHKNDATRRQR-ENSNS     643
ScYpl150w   IGL------------------KVAPELLLNEHIRIEEPRLKRFKSSISSEISQTSTGNY     602
              :                   *                 **  . ::.        .

HsMARK1     HGEQ---------------L-------RERRSVAYNGPPASPSHETGAFAHARRGTSTG     652
CaElk1      DGQSGKLERPQQLQLQHHRQLSQSQSLNTGLSTSGIDSPHTPPSAENSRFARTRPSSMIS     703
ScYpl150w   DSESAEN-------------------SRSI---SFDGKVSPPPI------RNRPLSEIS     633
            ..:.                             . :.  : *          : *  :  .

HsMARK1     IISKIT------------------------------------------------------     658
CaElk1      QISQLSKLSQMSTMLSESELDILDETDTMDDDDDYDDEVYESSINTSQDNKLTGPNATPS     763
ScYpl150w   QISNDTYISEYSTDGNNSSFKISDTIK-------------PSYIRK-------GSETTSQ     673
             **: :

HsMARK1     ----------------SKFVRRDPSE-------GEASGRTDTS-RSTSGEPKERDKEEG     693
CaElk1      SSNQGVKTSSKKRPTYRRG-VASDTSITSTPSASGGGAGGGS---GIISTIPKKKNSLS-     818
ScYpl150w   YS----ASSEKMTNGYGRKFVRRDLSIVSTASSTSERSSRTDSFYDITTATPV-------     722
                               *  * *         .  :.  .        :  *

HsMARK1     KDSKPRSLRFTWSMKTTSSMDPNDMMREIRKVLDANNCDYEQKERFLLFCVHGDARQDSL     753
CaElk1      ------------KLRSN---SSEDIS--------EDSYRFNDENM--PMGRSGSPDLGKK     853
ScYpl150w   ----------------------VT--------TDNRR--NKNN--NLKESVLPRFGTQ     746
                                  :           :.    :::     :        ..

HsMARK1     VQWEMEVCKLPRLSLNGVRFKRISG---------TS-----------------------     780
CaElk1      RSFRALQ--QPIPLVNG----------------TTSTSIAEKL----NDATKMPYVRAPS     891
ScYpl150w   RPWTGKR--TYTTSRHGKNARRSSKRGLFKITSSNTDSIIQEVSSSEEEDHNVIYSKGKG     804
              :              :*                .:

HsMARK1     ------------------------------------------------------------     780
CaElk1      PPIGFKYNAKANKKMMTTAINGNNNNNNN---------NQQQPPGYQD------------     930
ScYpl150w   LPTP----VLQTKGLIENGLNERDEEGDDEYAIHTDGEFSIKPQFSDDVIDKQNHLPSVK     860


HsMARK1     ------------------------------------------------     780
CaElk1      -------TKQVEDWIHNGTTGNTTTFTKSTIYQPVINEEEEENV-     967
ScYpl150w   AVATKRSLSEGSNWSSSYLDSDNNRRR---V-SSLLVEDGGNPTA     901
```

Supplemental Figure S1. Multiple sequence alignment of Cwr1 with orthologs from S. cerevisiae and H. sapiens.

Protein sequences for *C. albicans* Cwr1, *S. cerevisiae* Ypl150w, and human MARK1 were aligned using ClustalW.
