# Supplementary material for: The Cwr1 protein kinase localizes to the plasma membrane and mediates resistance to cell wall stress in Candida albicans
Source: mSphere. 2024 Nov 29;9(12):e00391-24. doi: 10.1128/msphere.00391-24 (PMC11656795; doi:10.1128/msphere.00391-24)
Supplement: Figure S2 — The cwr1Δ mutant grows invasively into agar containing alkaline pH, GlcNAc, or Spider medium. [file msphere.00391-24-s0002.pdf]

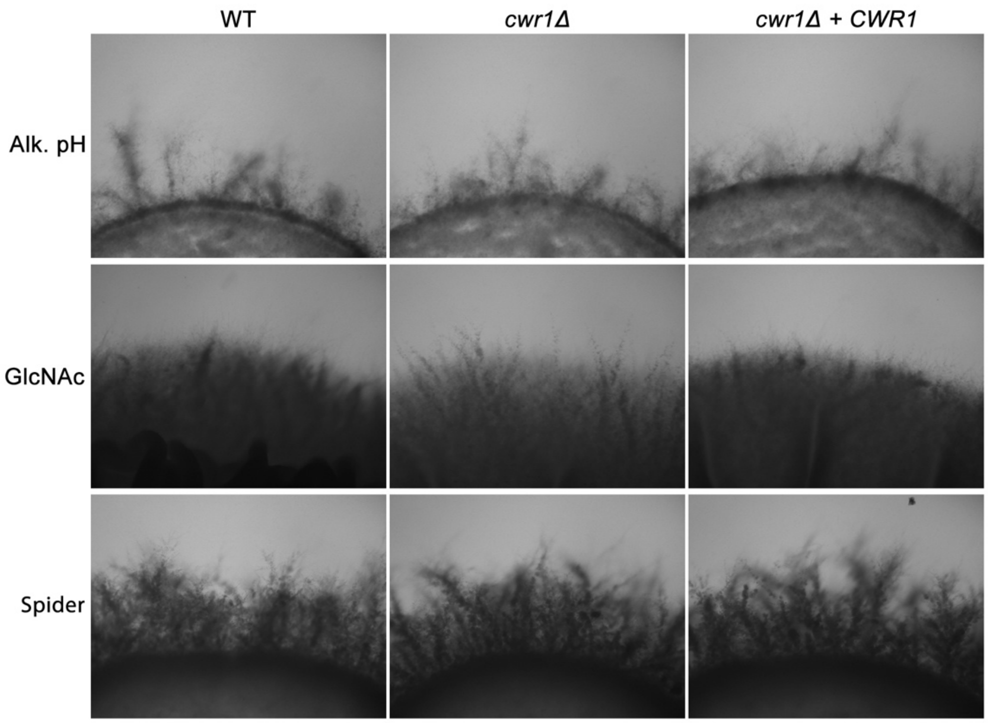

Supplemental Figure S2. The *cwr1*Δ mutant grows invasively into agar containing alkaline pH, GlcNAc, or Spider medium.

The indicated strains were spotted onto the surface of an agar plate containing the indicated medium and then incubated for 2 d at 37˚C. Media conditions included alkaline pH (100 mM BICINE pH 8), 20 mM N-acetylglucosamine (GlcNAc), and Spider medium (mannitol and nutrient broth).
