# Supplementary material for: The Cwr1 protein kinase localizes to the plasma membrane and mediates resistance to cell wall stress in Candida albicans
Source: mSphere. 2024 Nov 29;9(12):e00391-24. doi: 10.1128/msphere.00391-24 (PMC11656795; doi:10.1128/msphere.00391-24)
Supplement: Figure S3 — Comparison of Cwr1-GFP in cells grown in different media. [file msphere.00391-24-s0003.pdf]

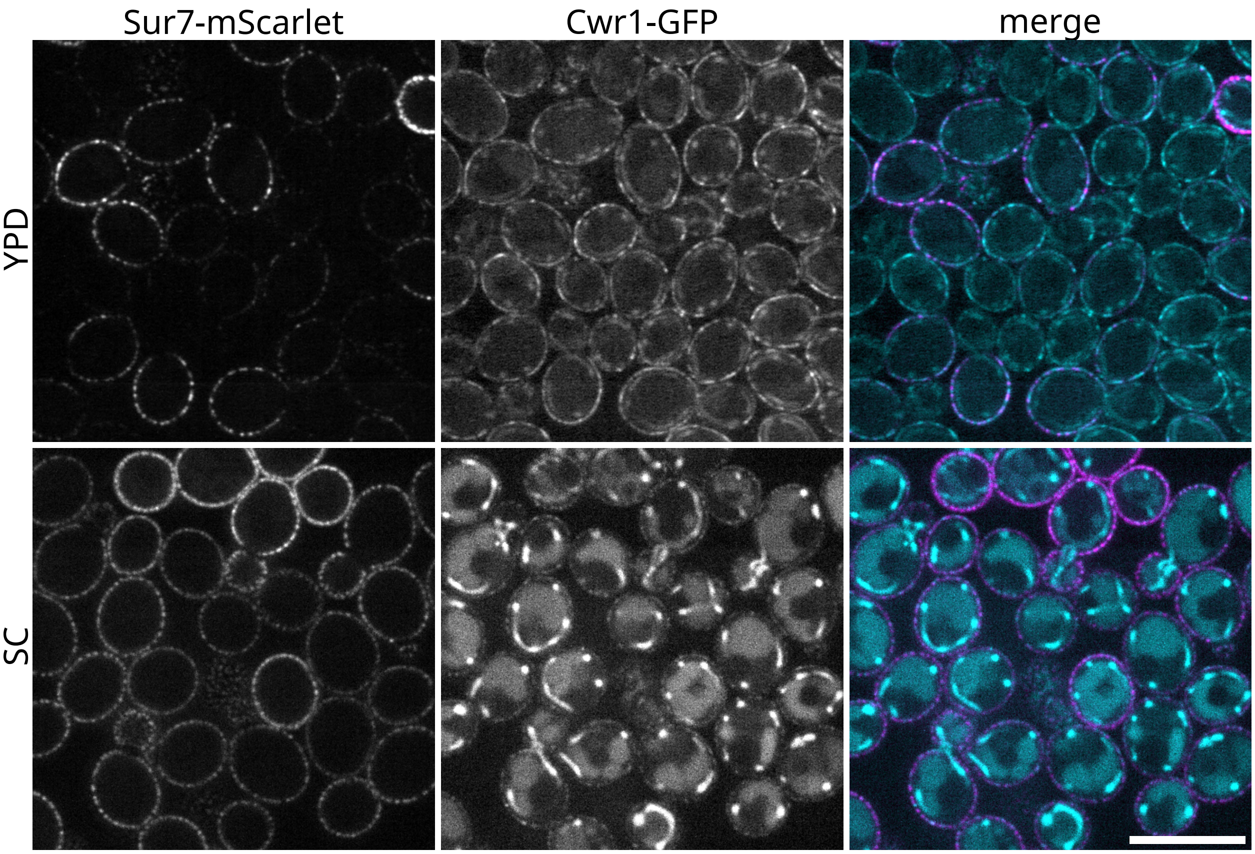

Supplemental Figure S3. Comparison of Cwr1-GFP in cells grown in different media. *C. albicans* cells carrying *CWR1-GFP* (cyan in the merged image) and *SUR7-mScarlet* (magenta in the merged image) were grown at 30˚C for 6 h in either YPD or synthetic complete (SC) media. The cells were then analyzed by confocal microscopy, where 10 consecutive images with 1 s acquisition time were recorded and the signal summed after drift and bleaching correction. Overlap of the signal from the two tags results in white color in the merged images. Scale bar: 10 µm. Note that while the plasma membrane Cwr1-GFP signal is higher and clearer in cells cultivated in YPD media, the cell-to-cell variation of Sur7-mScarlet signal is high, making the judgment of colocalization difficult.
