# Supplementary material for: The Cwr1 protein kinase localizes to the plasma membrane and mediates resistance to cell wall stress in Candida albicans
Source: mSphere. 2024 Nov 29;9(12):e00391-24. doi: 10.1128/msphere.00391-24 (PMC11656795; doi:10.1128/msphere.00391-24)
Supplement: Figure S4 — Quantitative analysis of Cwr1-GFP localization. [file msphere.00391-24-s0004.pdf]

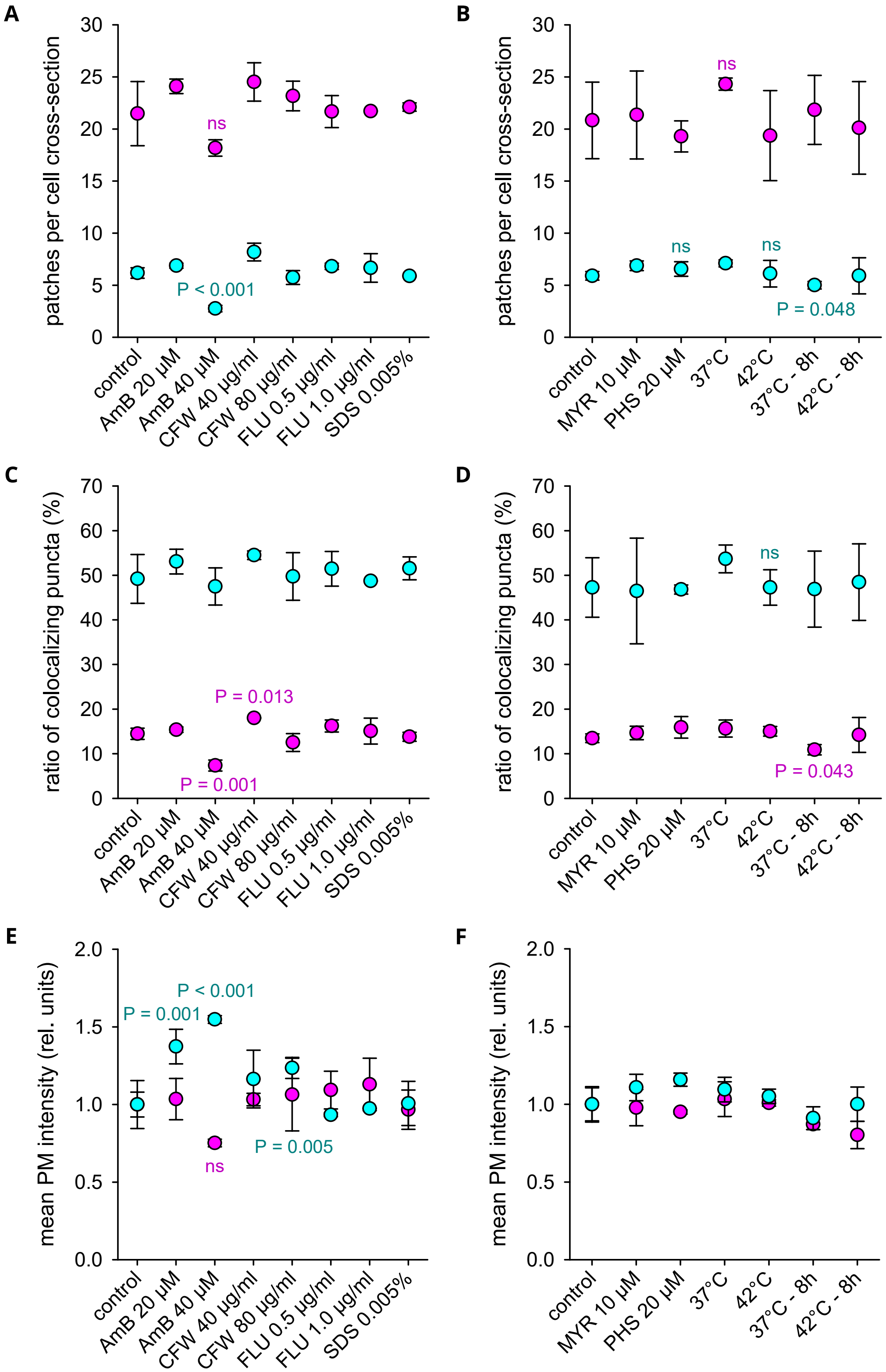

A
patches per cell cross-section
ns
P < 0.001
control
AmB 20 µM
AmB 40 µM
CFW 40 µg/ml
CFW 80 µg/ml
FLU 0.5 µg/ml
FLU 1.0 µg/ml
SDS 0.005%
B
patches per cell cross-section
ns
ns
ns
P = 0.048
control
MYR 10 µM
PHS 20 µM
37°C
42°C
37°C - 8h
42°C - 8h
C
ratio of colocalizing puncta (%)
P = 0.013
P = 0.001
D
ratio of colocalizing puncta (%)
ns
P = 0.043
E
mean PM intensity (rel. units)
P < 0.001
P = 0.001
P = 0.005
ns
F
mean PM intensity (rel. units)

Supplemental Figure S4. Quantitative analysis of Cwr1-GFP localization.

C. albicans cells expressing CWR1–GFP and SUR7-mScarlet were cultured and imaged as in Figures 7 and 8 and the following parameters were quantified from the microscopy images:

(A, B) Number of local accumulations (patches) per cell cross-section, obtained by watershed-based segmentation of raw microscopy images followed by masking of the plasma membranes (resulting in binary images) - see Methods for details.

(C, D) The ratio of Cwr1-GFP patches overlapping with Sur7-mScarlet patches (cyan) and Sur7-mScarlet patches overlapping with Cwr1-GFP patches (magenta), quantified from the overlap of the binary images used for the quantification of patches in A, B.

(E, F) Mean fluorescence intensity of Cwr1-GFP (cyan) and Sur7-mScarlet (magenta) in the plasma membrane (PM) relative to the untreated control.

Data are presented as mean ± SD from 3-5 biological replicates (100 to 300 cells in each replicate under each condition). P values ≤ 0.05 from multiple Student's t-tests (see Methods) are indicated in the graphs.
