# Supplementary material for: The Cwr1 protein kinase localizes to the plasma membrane and mediates resistance to cell wall stress in Candida albicans
Source: mSphere. 2024 Nov 29;9(12):e00391-24. doi: 10.1128/msphere.00391-24 (PMC11656795; doi:10.1128/msphere.00391-24)
Supplement: Table S1 — List of oligonucleotides used as primers to construct mutant strains and strains tagged with fluorescent proteins. [file msphere.00391-24-s0005.pdf]

Supplemental Table S1

Description: The following oligonucleotide primers were used in constructing the *cwr1*Δ deletion mutant, the strains in which CWR1 was tagged with GFP, and the strains in which SUR7 was tagged with the mScarlet fluorescent proteins, as described in the Materials and Methods.

Primers for ORF19.4518 (CWR1) Gene deletion Using CRISPR

1. 4518_sgRNA/F- GATGTGATGATAGAGTCAGAGTTTTAGAGCTAGAAATAGCAAGTTAAA
2. 4518_sgRNA/R- TCTGACTCTATCATCACATCCAAATTAAAAATAGTTTACGCAAGTC
3. Forward Primer (5' upstream of ATG)- CCGCGTAAATTTTAATCATTTTCAACTATACAATTCAACTTTTACTTCAAACTATTTTATTAGTT TAAACCTATATCCAAACCAGTGTGATGGATATCTGC
4. Primer 5 Reverse Primer (3' downstream of ORF) CATAGTTAGGCCGTTTACCTGGTAAATAAACTCATGCAGAAATTTATTGTTATGTTTTACTAT CATTACTAATATCAAGCATAGCTCGGATCCACTAGTAACG

ORF19.4518 Primers for creating complementing plasmid

5. F- GGTCATAGCTGTTTCCTGTGTGAAATTGTTATCCGCTCACTTCTATGTAAATGAGCGCGC
6. R- GGCCCCCCCTCGAGGTCGACGGTATCGATAAGCTTGATATTCATTACTAATATCAAGCAT

ORF19.4518 Detection Primers

7. 19.4518 det.F- GGGGTCAAATACGTTCATTC
8. 19.4518 det.F- ATCAACACCTTCAGCTTCTGGTGG
9. 19.4518 det.R- CTGTAAAGCTCGGAAACTTC

ORF19.4518 GFP tagging

10. ORF 19.4518- GFP-F GTACTACAGGAAATACAACTACGTTTACCAAATCAACAATTTATCAACCGGTTATCAATGAAG AGGAAGAAAATGTAGGTGCTGGCGCAGGTGCTTC
11. ORF 19.4518- GFP-R- AAATGAGACACATAGTTAGGCCGTTTACCTGGTAAATAAACTCATGCAGAAATTTATTGTTAT GTTTTACTATCATTCTGATATCATCGATGAATTCGAG
12. 19.4518 T329-GFP Fwd. CCAGTGGTAGTGGAGATGCCGAATCAATAATATCGATTAATCAACATTACAATACAGTGGAT AGACCATTTGAGGCCAAGGGTGCTGGCGCAGGTGCTTC
13. 19.4518.T527-GFP Fwd. CCGCTGATTTGAATACGCCTTTACGTCGTACTGTATCATTTGTTCCTGATGGAAGAAGGCTA TCACAAGTAATATCATCAGGTGCTGGCGCAGGTGCTTC
14. 19.4518.T742-GFP Fwd. CTATGTTGTCGGAATCTGAGTTGGATATTCTTGACGAAACCGATACAATGGACGATGATGAT GATTATGATGATGAAGTAGGTGCTGGCGCAGGTGCTTC
15. ORF19.4518FwdCk1 GFP verification primer CCTCCAGGTTATCAAGACAC
16. ORF19.4518FwdCk2 GFP verification primer. CCATATGTTCGAGCTCCATC
17. ORF19.4518FwdCk3 GFP verification primer GATCATGTCCCCATTGACGT
18. ORF19.4518FwdCk4 GFP verification primer GCTTGTTTGTTTGGACTTGG
19. ORF19.4518FwdCk5 GFP verification primer CTTGGTTCAGTTCAGTTTGG

SUR7-mScarlet primers

20. Sur7mScarletFwd GATGAAGAAAACACAGGCGGTATTAGATTCTTCAAAATCAAAAGAAACCAAAAAGTTTCCGA TGATGAATCAGTA GGTGGTAGTGGTATGGTTTCTAAAG

21. Sur7mScarletRvs GTAAGGAATAATGTCAGACTCAAATTGATGGTTTGCATATACCAATTGGTATATTTAATATAC GATTTCTTAAT GGCGGCCGCTCTAGAACTAGTGGATC
